# Application of Enhanced Sampling Monte Carlo Methods for High-Resolution Protein-Protein Docking in Rosetta

**DOI:** 10.1371/journal.pone.0125941

**Published:** 2015-06-08

**Authors:** Zhe Zhang, Christina E. M. Schindler, Oliver F. Lange, Martin Zacharias

**Affiliations:** 1 Physik-Department T38, Technische Universität München, James-Franck-Str. 1, 84748 Garching, Germany; 2 Biomolecular NMR and Munich Center for Integrated Protein Science, Department Chemie, Technische Universität München, Lichtenbergstr. 4, 85748 Garching, Germany; Consiglio Nazionale delle Ricerche, ITALY

## Abstract

The high-resolution refinement of docked protein-protein complexes can provide valuable structural and mechanistic insight into protein complex formation complementing experiment. Monte Carlo (MC) based approaches are frequently applied to sample putative interaction geometries of proteins including also possible conformational changes of the binding partners. In order to explore efficiency improvements of the MC sampling, several enhanced sampling techniques, including temperature or Hamiltonian replica exchange and well-tempered ensemble approaches, have been combined with the MC method and were evaluated on 20 protein complexes using unbound partner structures. The well-tempered ensemble method combined with a 2-dimensional temperature and Hamiltonian replica exchange scheme (WTE-H-REMC) was identified as the most efficient search strategy. Comparison with prolonged MC searches indicates that the WTE-H-REMC approach requires approximately 5 times fewer MC steps to identify near native docking geometries compared to conventional MC searches.

## Introduction

Protein-protein interactions are integral to many mechanisms of cellular activities, ranging from enzyme catalysis and inhibition to signal transduction and gene regulation. Atomic-level structural knowledge is essential to understand the function of protein-protein complexes in biological processes. However, experimental structure determination of protein-protein complexes is often difficult and for many interactions the corresponding complex structures are lacking [1,2]. Computational protein-protein docking methods can provide structural models of protein-protein interactions where experimental data is absent, of low-resolution or too sparse. Besides providing valuable structural biology information, high-resolution protein-protein docking can also help to explain binding affinities and specificities, the nature of the binding free energy funnel and effects of mutations. Furthermore, these techniques are essential for computational protein-protein interface design of the design of non-natural complexes [3–7]

Docking programs often employ a two-stage protocol [8,9]. First, the space of putative docking geometries is sampled broadly, keeping the partner structures rigid, which corresponds to six degrees of freedom. Second, structures are refined in one or multiple steps, typically employing partner structures at atomic resolution allowing for conformational changes of side chains and possibly also of the protein main chain. For the rigid-body stage, the application of grid-based fast Fourier transformation [10] or geometric hashing [11] can accelerate the search [8,9]. Alternatively, the search can also be performed efficiently at reduced resolution using a coarse-grained model of the protein structures [12,13]. Both, at the rigid-body search stage or during refinement, Monte Carlo (MC) methods can be very helpful [14–18].

For high-resolution refinement, most protocols require a starting configuration that is already fairly close to the native structure. The likely reason for this strong dependence on the starting structure is the energy landscape’s ruggedness, which frustrates the sampling and renders the global energy minimum hard to reach [19]. Accordingly, the rationale behind typical two stage docking refinement protocols is that the initial docking predictions is likely to generate at least one structure close to the native. A justification of this assumption is given by the general hypothesis that the native conformation coincides with the global energy minimum at the bottom of a broad basin in a rugged energy landscape [20,21]. However, since initial docking stages often use a simplified energy function, the initial docking stage is routinely misled and does not actually produce sufficiently many near-native candidates for the subsequent refinement stage, especially when there is an alternative binding site with larger buried surface [14].

Focus of this work is to improve the sampling for high-resolution docking based on the MC approach. In the MC method, random translational and rotational moves or conformational changes of the partner structures are applied on the configuration in a step-wise manner using the Metropolis criterion for acceptance of a move. Advantages of the MC method are the generation of a physically relevant canonical ensemble of docking configurations, use of arbitrary energy functions that can contain discontinuities and for the possibility to include various levels of structural flexibility. However, an exhaustive high-resolution sampling of the conformational space with the MC method can be computationally demanding. In general, the docking success of MC docking is limited by the sampling of putative complex geometries and by the accuracy of the energy function used for scoring predicted complexes.

Parallel tempering or replica exchange techniques promise to overcome these challenges and have received wide-spread interest in recent years [22–25]. The general idea of parallel tempering is to simulate the system with M replicas at different temperatures and to frequently exchange configurations between neighboring replicas. The high temperature replicas sample broadly, whereas the low-temperature replicas allow precise exploration of deep energy minima. Due to the frequent exchanges between the (hot) broad sampling regime and the (cold) annealing regime, configurations are less likely to get trapped in local minima. A generalization of temperature replica exchange is to vary the Hamiltonian (H-REMC) among replicas [26], which allows, for instance, to blend between a smoothed van der Waals potential and a realistically hard formulation to allow overcoming of sampling barriers in molecular dynamics simulation [27,28]. For Rosetta, previous studies also showed that softening the Lennard-Jones repulsive term is beneficial and better suited for side-chain modeling and prediction [29,30]. Of course, it is possible to combine variation of temperature and Hamiltonian in multi-dimensional replica exchange approaches [31–35]. A bottleneck in using replica exchange is that to cover the same parameter range (temperature, or smoothness) the number of replicas required increases quickly with the number of degrees of freedom sampled. This is due to the fact, that to achieve efficient exchange between replicas, a substantial overlap between sampled energy levels of neighboring replicas is required [33,36].

Metadynamics is another popular enhanced sampling method, in which sampling is facilitated by a history-dependent biasing potential. It is constructed as the sum of Gaussian functions deposited along the trajectory in the collective variable space [37,38]. Choosing energy as collective variable gives rise to the so-called well-tempered ensemble (WTE) with much larger fluctuations in the sampled energies than the canonical ensemble [39]. This property of the WTE can be exploited to overcome the major bottleneck of temperature replica exchange discussed above. Since, the overlap of the energy distribution between neighboring replicas controls the exchange efficiency, using WTE drastically reduces the number of replicas required [39,40].

In the present study we have compared the efficiency of a standard MC protocol for high resolution protein-protein docking using *RosettaDock* and various extensions based on advanced sampling techniques. In particular, we tested four different protocols, standard Monte Carlo (MC), Temperature Replica Exchange Monte Carlo (REMC), well-tempered ensemble temperature Replica Exchange Monte Carlo (WTE-REMC), and well-tempered ensemble two dimensional Hamiltonian Replica Exchange Monte Carlo (WTE-H-REMC). The approaches were systematically evaluated on protein-protein complexes using unbound partner structures and starting in each case from the same starting placements. Overall best performance was achieved with the WTE-H-REMC method at the same computational cost compared to the alternative protocols.

## Methods

### Energy scoring function and starting structure generation

**Fig 1:**
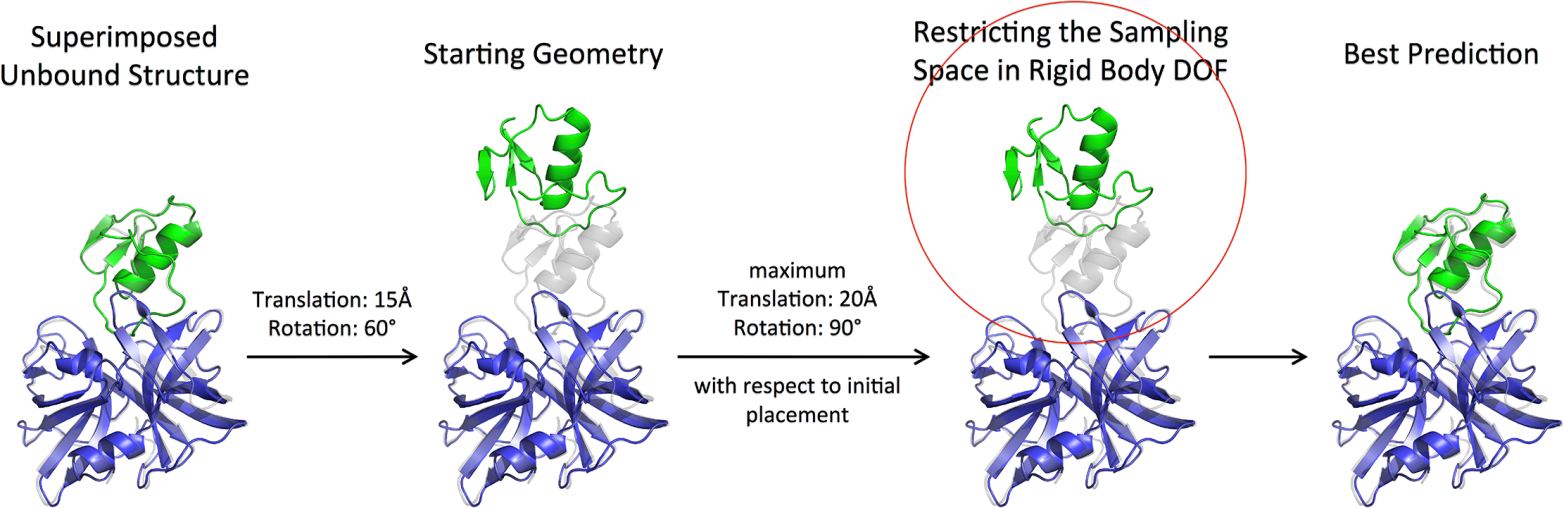
Docking refinement conditions. Each docking starting geometry was generated by an initial random translation of one unbound partner from the geometry in the complex by 15Å and random rotation of 60° (compare green displaced and grey cartoon representations). During the docking search translation and rotation of one partner with respect to the other was restricted relative to the starting geometry by 20Å and 90° (indicated by red circle), respectively.

The standard all-atom energy function for *RosettaDock* as given by the weight-set *docking* [41] was used in all docking protocols. The docking energy function consists of van der Waals attractive and repulsive interactions, an implicit solvation term, hydrogen-bonding energies, a statistical residue-residue pairwise interaction term, a rotamer probability term and a pairwise electrostatic energy term [18,42]. For each target, the different docking simulation protocols were started from the same initial protein partner arrangements. The start geometries were based on unbound partner structures and one partner was initially separated relative to the position in the complex in a random direction by 15 Å and a random rotation by 60° relative to the bound geometry. Only geometries without steric overlap between partners were accepted. The ligand RMSD (L_rmsd, root mean square deviation of the mobile protein after best superposition of the receptor protein onto native complex structure) from the respective bound complex for all the targets was on average ~18 Å with slight variation depending on the shape and size of the binding partners (Fig 1). The initial placement corresponds to a scenario where the binding region is approximately known.

### Restricting the sampling space in rigid body degrees of freedom

For rigid body moves, random translations drawn from Gaussian distribution are performed along all three axes, and the axis-angle notation is used to represent rotations [14]. In order to perform a local search in the vicinity of the starting geometry, the sampling space in the rigid body degrees of freedom was restricted with respect to the initial input conformation by a maximum translation of 20Å and maximum rotation of 90° (this exceeds the maximum displacement of the starting structure from the bound configuration, see previous paragraph and illustration in Fig 1). To avoid dissociation of the two binding partners, we also applied an encounter constraint, which acts on the distance between the center of mass of the two binding partners and only penalizes the sampled geometries if the two binding partners are too far apart [14].

### General settings

We have combined enhanced sampling techniques with Monte Carlo (MC) method to sample protein-protein docking with atomistic representation, and tested four protocols within Rosetta including standard MC, Temperature Replica Exchange Monte Carlo (REMC), well-tempered ensemble temperature Replica Exchange Monte Carlo (WTE-REMC), and well-tempered ensemble 2-dimensional Hamiltonian Replica Exchange Monte Carlo (WTE-H-REMC). In those docking approaches, rigid body displacements and side-chain optimization are accomplished by the rigid body mover UnbiasedRigidBodyPerturbNoCenterMover, and sidechain movers including JumpRotamerSidechainMover, PerturbRotamerSidechainMover and PerturbChiSidechainMover. Those movers are applied under the control of the Metropolis-Hastings framework. For each move, the MetropolisHastingsMover randomly applies one out of the four movers based on their sampling weights. Mover step size for UnbiasedRigidBodyPerturbNoCenterMover and PerturbChiSidechainMover are drawn from random Gaussian distributions. In the protocols with replica exchange, the magnitude for mover step size and sampling weight were modulated according to the replica level during initialization such that in the lower levels more frequent side-chain moves and fewer small rigid body moves were applied and in the higher levels less frequent side-chain moves and larger rigid-body moves. The magnitude of the step size and sampling weight were, however, kept fixed along the simulation in each replica. All the settings for the reference replica were made exactly the same as used in the standard MC protocol, and we denote these settings as reference settings. If not indicated otherwise, for all the protocols and on each target 2x10^6^ MC steps were employed. Snapshots are taken and stored every 1,000 steps. In the REMC protocols exchanges were attempted every 1,000 MC steps.

### Monte Carlo and Asynchronous Parallel Tempering protocol

For the standard MC docking protocol, 25 trajectories are run with temperature set to 0.15. At the end, about 25 x 2,000 sampled structures were collected for each target. The step size of translation and rotation for rigid-body moves are drawn from normal distributions with small mean value of 0.1Å and 1°. The sampling weights for UnbiasedRigidBodyPerturbNocenter, JumpRotamerSidechain, PerturbRotamerSidechain and PerturbChiSidechain are set to 0.5, 4, 6 and 10, respectively.

For the parallel tempering replica exchange (REMC) protocol, 13 temperature levels were drawn from geometric progressions ranging from 0.15 (reference) to 0.31. Two trajectories with the 13 replicas are run for each target. Exchanges were attempted between neighbor replicas every 1,000 steps. For all targets, good exchange rates (between 25% and 69% with median value 49%) are achieved and no further target dependent optimization was required. In replica exchange, it is common that the speed of each replica is not exactly the same. To avoid that the faster replica wait for the slower partner, we used an asynchronous exchange scheme. That is the faster replicas can perform more steps instead of waiting for its partner to reach the pre-defined exchange stride. Finally, the simulation will finish as soon as the slowest replica has reached the required step number.

### Well-Tempered Parallel Tempering and Hamiltonian replica exchange protocol

We applied the well-tempered ensemble (WTE) technique with parallel tempering replica exchange Monte Carlo using a value of 5 for the tunable factor *γ* and reduced the temperature levels from 13 to 5 with the same range. The bin size for well-tempered ensemble technique to collect the history-dependent bias energy is set to two units of Rosetta docking energy. The resulting exchange rates are between 20% and 55% with median value 37%. For the WTE-H-REMC protocol we took advantage of the splitting of the van der Waals interactions into attractive and repulsive components in *RosettaDock*. It is represented with a modified Lennard-Jones 6–12 potential which includes a linear extrapolation in the repulsive part below the threshold of 0.6*σ*_*ij*_, where *σ*_*ij*_ is the sum of the atomic radii of atoms *i* and *j*. The atomic radii and energy well depth are taken from the CHARMM19 parameter set [13,18,43], and we denote this as "hard-rep". For the standard "soft-rep" in Rosetta, the atomic radii were either held fixed or scaled by a factor of 1.07 (typically for non-polar atoms) from the hard-rep radii, and the "switch point" for the linear extrapolation was selected empirically [29]. In the WTE-H-REMC protocol, we applied a 2-dimensional replica exchange, with the temperature as variable in the first dimension, and used the scaling factor for the soft Lennard-Jones repulsive term as the second dimension. The scaling factor allows linear interpolation of atomic radii and switch point between the hard-rep and soft-rep potentials (see above). In the Hamiltonian scaling dimension, we used five levels: hard_rep, soft 50%, soft 55%, soft 60% and soft 65%. In the temperature dimension five temperatures between 0.15 and 0.3 were used (in arbitrary units depending on the scaling of the Rosetta score), yielding a total of 25 replicas. Exchange between neighboring replicas is attempted every 1,000 steps along the two dimensions. Well tempered ensemble technique was applied again to improve the exchange rate with tunable factor *γ* = 5. The bin size for well-tempered ensemble technique to collect the history-dependent bias energy is set to two units of the Rosetta docking energy. Note, that each replica accumulated separate history-dependent biasing potentials depending on the individual sampling history. Replica exchange rate ranged between 14% and 56% in the temperature dimension with median value 32%. In the scaling dimension, it increased along the shifting from soft to hard repulsive interaction, and ranged between 12% and 99% with median value 39%.

### Implementation in Rosetta

Previously, we implemented replica exchange within the general Metropolis-Hastings framework of the Rosetta3 software package [14]. The replica exchange module is accessible through the RosettaScripts interface and can be combined with any conformational moves that are implemented as children of the ThermodynamicMover class. For rigid-body docking refinement, we have applied a rigid-body mover UnbiasedRigidBodyPerturbNoCenterMover, and sidechain movers including JumpRotamerSidechainMover, PerturbRotamerSidechainMover and PerturbChiSidechainMover. For detailed balance, UnbiasedRigidBodyPerturbNoCenterMover performs unbiased rotational and translational perturbations in the restricted space as described in the previous section, and sidechain movers provide the proposal density of the perturbation through implementation of the abstract virtual function compute_proposal_density() in the ThermodynamicMover interface. The acceptance of a move is decided by the Metropolis criterion [44].

Side-chain motion is applied on one randomly selected residue (among all the residues and all the residue types but Proline) each time. Continuous sampling of side chain chi angles are used instead of fixed rotamers in all the three sidechain movers. The angles are chosen according to the Dunbrack rotamer library 2010 probabilities [45]. PerturbChiSidechainMover does a perturbation on the side chain *chi* angles, either uniformly distributed or Gaussian distributed with a given magnitude. For JumpRotamerSidechainMover and PerturbRotamerSidechainMover, a rotamer is first selected randomly or selected such that it has the highest probability of proposing the old *chi* angles according to the Dunbrack rotamer library probabilities, respectively, then individual *chi* angles are chosen using Gaussian distributed random angles with the means and standard deviations from the Dunbrack rotamer library.

The well-tempered ensemble technique is implemented into the framework of MetropolisHastings as ThermodynamicObserver. It is applied with a certain time interval (here in the test stride = 1) and deposits the Gaussians to the bias energy with height of

$$W=\omega e^{-\left[V\left(s,t\right)/\Delta T\right]}\tau_{G}$$

where *τ*_*G*_ is the time interval or stride number, *V*(*s*, *t*) is the old bias energy in the energy bin where the current decoy's energy has dropped into, *ω* represents the initial bias deposition rate and Δ*T* = (1 − *γ*)*T*, in which *γ* represents the tunable factor and *T* is the temperature in the simulation [37,39,40,46]. When well-tempered ensemble technique is applied, acceptance of a move or an exchange attempt is decided based on the total energy, which is the sum of the force field energy and the bias energy. For final analysis, only the force field scoring energy was used.

### Construction of a benchmark

The four protocols were first tested on 10 unbound targets (Table 1) from the benchmak4.0 set [47,48] with reasonable energy funnels using the RosettaDock scoring force field. This was checked by generating 1000 decoys with standard RosettaDock full protocol starting from the bound docking geometry (using unbound structures). These 10 targets belong to the group of “rigid body” docking cases with small changes of side chains associated with complex formation (according to the classification of the protein docking benchmark4.0 collection [47,48]). In addition, the standard MC protocol and the WTE_H_REMC protocols were also tested on another 10 unbound targets including one "difficult" (1JK9) and two "medium difficulty" (1MQ8 and 2CFH) targets (Table 1). The number of residues of the 20 targets range between 122 and 409.

**Table 1:** Test complex structures and partner structures.

Complex	Cat.	Difficulty	Partner I	Nres 1	Partner II	Nres 2	RMSD (Å)	DASA (Å)
1EAW_A:B	E	rigid	1EAX_A	241	9PTI_	56	0.54	1866
1GCQ_B:C	O	rigid	1GRI_B	66	1GCP_B	56	0.92	1208
1KTZ_A:B	O	rigid	1TGK_	105	1M9Z_A	82	0.39	989
1PPE_E:I	E	rigid	1BTP_	223	1LU0_A	29	0.44	1688
1S1Q_A:B	O	rigid	2F0R_A	141	1YJ1_A	69	0.98	1288
2AYO_A:B	O	rigid	2AYN_A	337	2FCN_A	72	1.39	3027
2SNI_E:I	E	rigid	1UBN_A	274	2CI2_I	64	0.35	1628
3D5S_A:C	O	rigid	1C3D_A	294	2GOM_A	61	0.56	1620
3SGQ_E:I	E	rigid	2QA9_E	185	2OVO_A	51	0.39	1211
7CEI_A:B	E	rigid	1UNK_D	127	1M08_B	87	0.7	1384
1AY7_A:B	E	rigid	1RGH_B	96	1A19_B	89	0.54	1237
1H9D_A:B	O	rigid	1EAN_A	125	1ILF_A(1)	114	1.32	2121
1HE1_C:A	O	rigid	1MH1_	176	1HE9_A	128	0.93	2113
1JK9_A:B	O	difficult	1QUP_A	219	2JCW_A	153	2.51	2130
1MQ8_A:B	O	medium	1IAM_A	184	1MQ9_A	171	1.76	1253
1RV6_VW:X	O	rigid	1FZV_AB	189	1QSZ_A	92	1.09	1626
1YVB_A:I	E	rigid	2CHU_A	241	1CEW_I	108	0.51	1743
2CFH_A:C	O	medium	1SZ7_A	156	2BJN_A	137	1.55	2384
2OUL_A:B	E	rigid	3BPF_A	236	2NNR_A	107	0.53	1933
2SIC_E:I	E	rigid	1SUP_	275	3SSI_	107	0.36	1617

cat: Complex category labels: E = Enzyme/Inhibitor or Enzyme/Substrate O = Others.

RMSD: RMSD of Ca atoms of interface residues calculated after finding the best superposition of bound and unbound interfaces.

DASA: Change in Accessible Surface Area upon complex formation.

### Analysis of docking results and computational efficiency

The sampled docked complex were analyzed according to ligand RMSD (L_rmsd) and fraction of native contacts (*f*_nat_), as defined in CAPRI [49] using the bound complexes as references. To evaluate the capacity of the methods to sample near-native decoys, we calculated the fraction of CAPRI medium (**, *f*_*nat*_ ≥ 0.5 & L_rmsd > 1*Å* or 0.3 ≤ *f*_*nat*_ < 0.5 & L_rmsd ≤ 5) or high (***, *f*_*nat*_ ≥ 0.5 & L_rmsd ≤ 1*Å*) quality decoys in the collected results [50]. To evaluate the agreement between generated complexes with closest L_rmsd and best *f*_nat_ compared to the bound complex. To evaluate the scoring energies the interaction score (I_sc) was used which is computed by subtracting the all-atom energy of non-interacting partners from the all-atom energy of the interacting partners in the complex. To compute the energy of non-interacting partners the two binding partners are moved far away from each other while keeping all internal degrees of freedom fixed. To investigate the efficiency of optimizing the scoring energies, we calculated for a given MC step number the average difference of the sampled best score (up to the selected MC step number) and the final most favorable score.

## Results and Discussion

**Fig 2:**
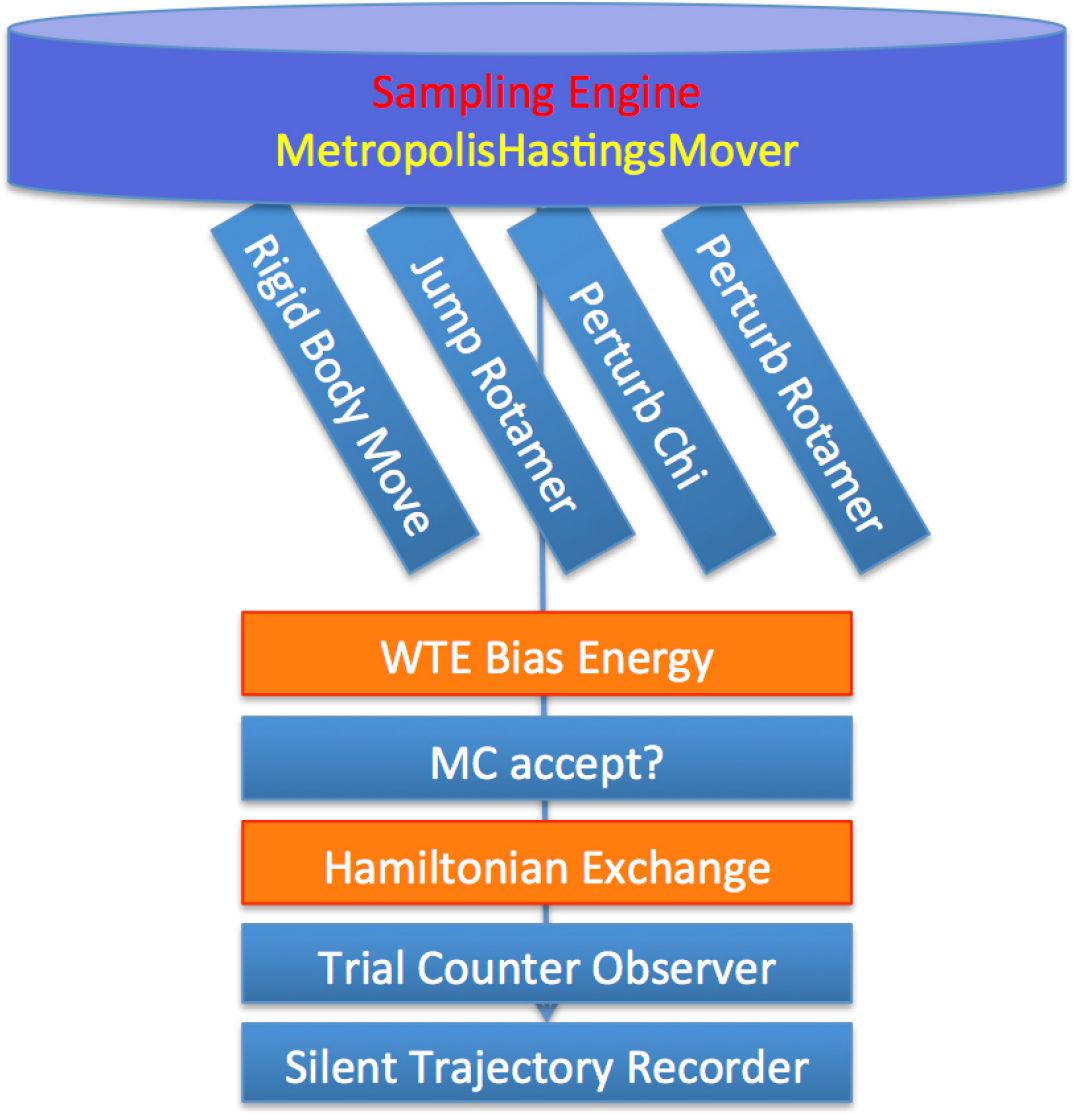
Workflow represented by combination of Rosetta modules and setup of the four docking protocols. The modules in orange, representing the enhanced sampling techniques of replica exchange and well-tempered ensemble, are only applied in the combined protocols.

Monte Carlo docking simulations are frequently used to perform protein-protein docking searches or for the refinement of predicted complexes at atomic resolution including limited conformational changes of the partner structures [14–17]. In recent years, enhanced sampling methods to improve the MC search efficiency have been developed. In order to test the performance of such improvements we compare the application of standard MD, parallel tempering REMC, well tempered replica exchange (WTE-REMC), and well tempered ensemble combined with 2-dimensional temperature and Hamiltonian replica exchange (WTE-H-REMC) to a set of protein-protein complexes in unbound partner conformation. In each case the MC moves included rigid body translation and rotation as well as side-chain moves (illustrated in Fig 2). For each protocol the same start configuration was used corresponding to a random arrangement of one mobile partner placed approximately 15 Å away from the bound complex geometry (see Methods for details). In case of the replica exchange methods only configurations in the reference replica were retained, resulting in approximately 25x2,000 decoys for standard MC protocol (with 2x10^6^ MC steps), 2x2,000 for REMC protocol, 5x2,000 for WTE-REMC protocol and 1x2,000 for WTE-H-REMC protocol, respectively. On a 2.6 GHz AMD Opteron Processor (12 cores), 2x10^6^ MC steps take between 4.5–20 hours. The sampled docking solutions were analyzed in terms of deviation from the known complex geometry (using the root mean square backbone deviation of the mobile ligand partner protein from the bound complex after best superposition of the receptor protein onto the bound complex: L_rmsd) and interaction score (I_sc).

**Fig 3:**
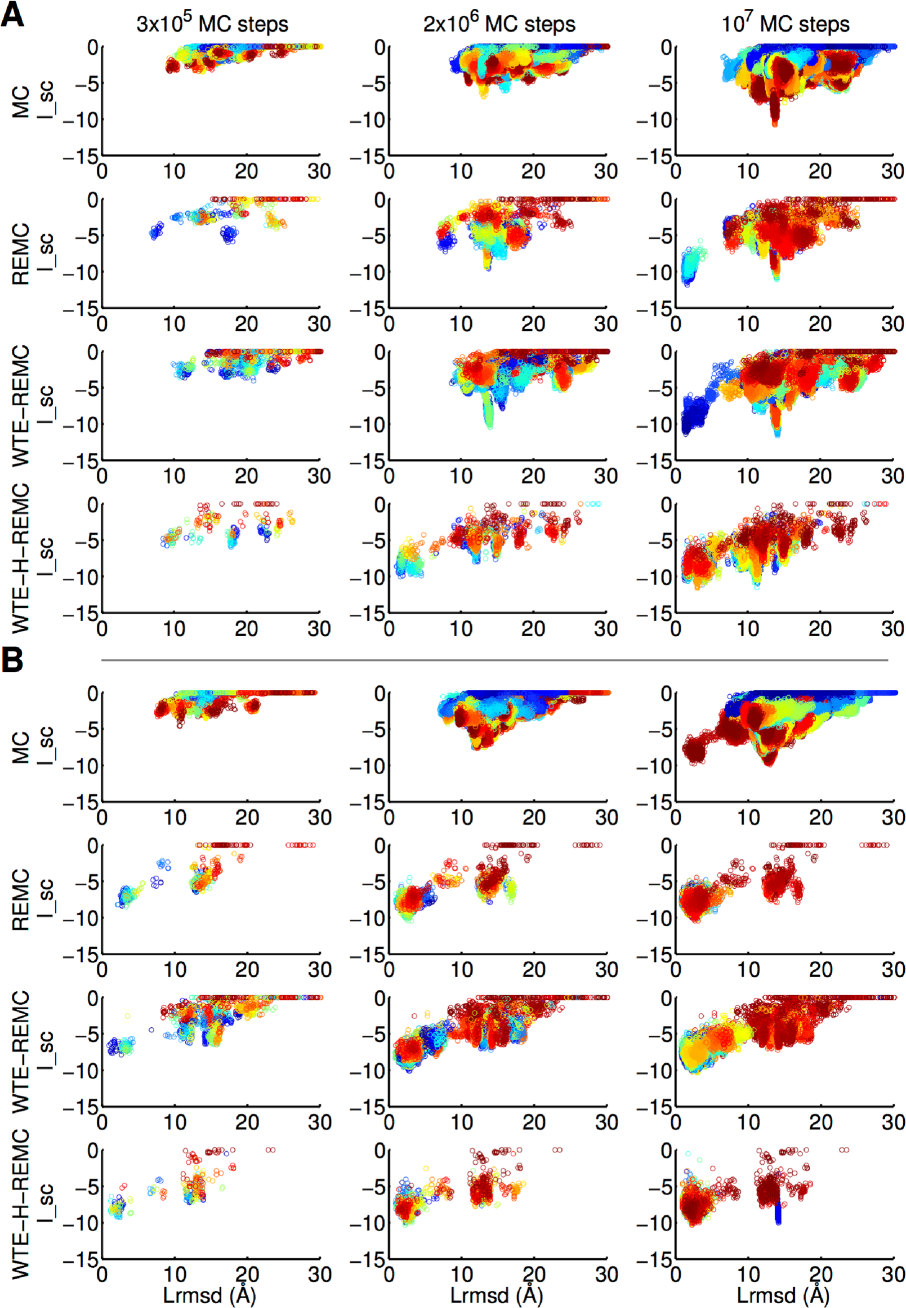
Scatter plot of interaction score I_sc (Rosetta units) vs. L_rmsd (Å) for the four docking refinement protocols and two representative targets, 1EAW (A) and 3SGQ (B). The protocol is indicated on the left for each row of plots. The snapshots number is color-coded, that means blue and red dots corresponding to decoys sampled at the beginning and the end of the docking searches in each panel, respectively. The three columns of plots indicate the result after different simulation lengths (indicated on top of each column).

The protocols were first tested on 10 benchmark targets of the “rigid body” category (with only limited side chain changes upon complex formation, see Methods for details). We consider the sampling of medium or high-quality solutions (CAPRI **/*** solutions, defined in Methods section) according to the CAPRI criteria as successful docking refinement. The evolution of the sampling in terms of L_rmsd and I_sc scoring is showcased for two representative targets (pdb1EAW and pdb3SGQ) in Fig 3. For the pdb1EAW-target all methods sample progressively lower (more favorable) force field scores with increasing number of MC steps. However, for the first example only the WTE-H-REMC protocol samples docking solutions with L_rmsd < 5 Å after 2x10^6^ MC steps. Only after 10^7^ MC steps all techniques except the standard MC technique sample near-native solutions (Fig 3A). For the second example (pdb3SGQ) the MC technique successfully samples solutions with Lrmsd < 5 Å only after 10^7^ MC steps whereas all three advanced sampling techniques reach near-native solutions already after 3x10^5^ MC steps (Fig 3B). Qualitatively similar trends were observed for all other test cases (see Figures A-C in S1 File).

**Fig 4:**
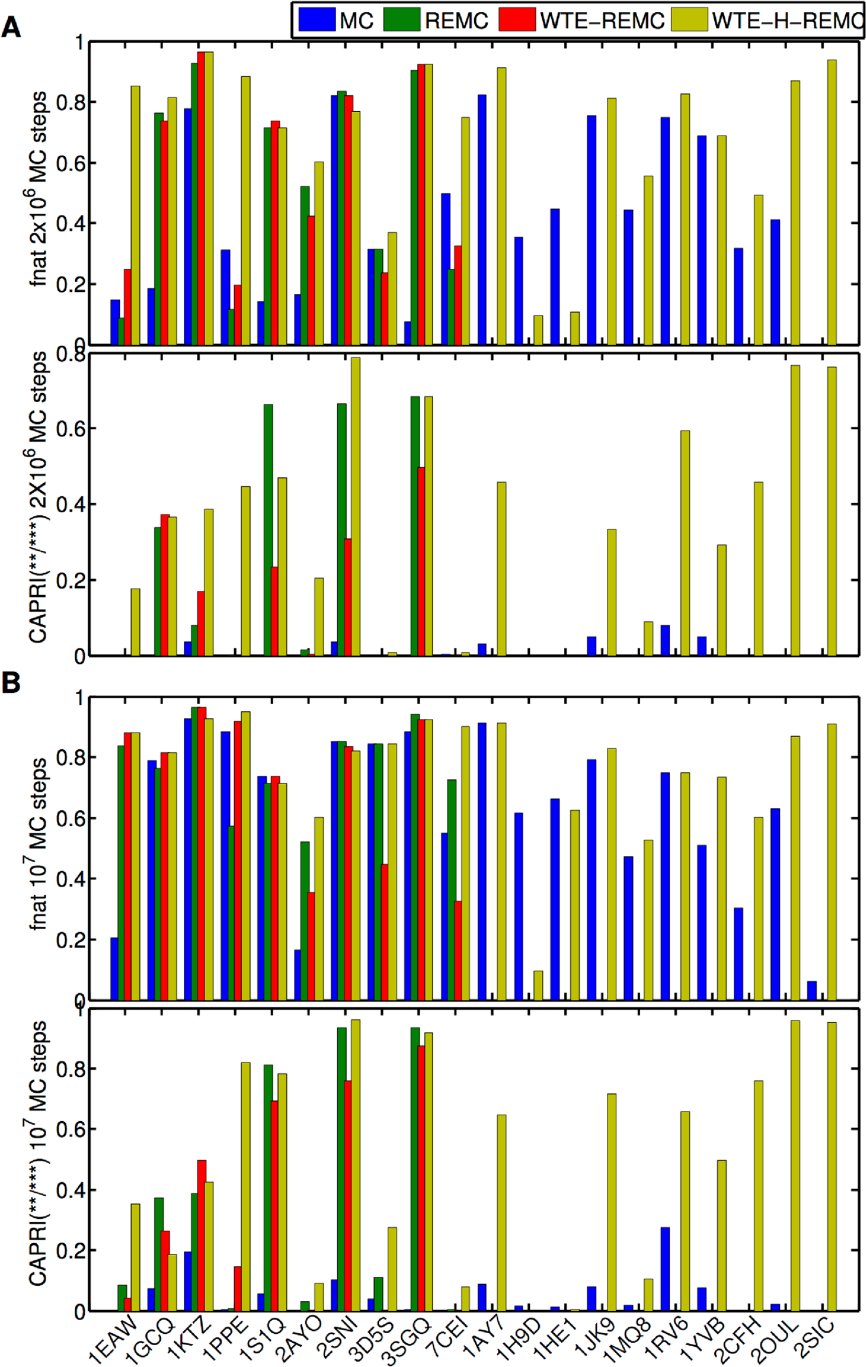
Agreement between sampled docking geometries and the corresponding bound complex. (A, upper panel) Highest fraction of native contacts (*f*_nat_) found in the top 10 decoys (according to L_rmsd) sampled in each protocol (2x10^6^ MC steps). (A, lower panel) Fraction of CAPRI medium and high quality complexes found for each target and each protocol (the protocols MC, REMC, WTE-REMC and WTE-H-REMC are indicated by different colors). (B) same as in (A) but for the docking refinement runs with 10^7^ MC steps.

A quantitative comparison of the docking refinement solutions in terms of the fraction of native contacts (*f*_nat_) indicates that the WTE-H-REMC method succeeded in all 10 cases in sampling near-native docking solutions with very high quality (Fig 4A). In contrast, the other protocols succeeded only in 3 (MC) or 7 (REMC and WTE-REMC) of the first 10 cases (Fig 4A). Note, that near-native docking solutions are also the best scoring solutions in several but not all docking test cases (e.g. for targets 1KTZ, 3D5S and 7CEI docking solutions with Lrmsd > 10 Å give I_sc scores lower than the solutions closest to the bound docking geometry, Figures B,C in S1 File). The results on the first 10 test cases indicate that the WTE-H-REMC enhanced sampling protocol showed the best performance. For a second test set of 10 targets (including also targets of the “medium” and “difficult” category, see Table 1), only the standard MC and the WTE-H-REMC protocols were compared. Again, the WTE-H-REMC protocol gave better docking results in 5 cases (IJK9, 1MQ8, 2CFH, 2OUL, 2SIC) with lower final I_sc scores and Lrmsd (Fig 4A, Figures D-F in S1 File) compared to the standard MC-method. However, in two cases (1H9D and 1HE1) the standard MC–method reached configurations closer to the bound form compared to the WTE-H-REMC technique. Note, that especially in these cases the score of near-native docking solutions was higher (less favorable) than for alternative docking geometries (Figures E, F in S1 File). Since the search techniques optimize the score (and not deviation from bound structure) it may explain the failure of the WTE-H-REMC technique in these cases.

To check if slower convergence to reach low energy docking solutions was the main reason for the failure of some protocols to reach near-native docking solutions, we increased the trajectory length to 10^7^ MC steps. Indeed, the success of MC, REMC, and WTE-REMC protocols to reach near-native docking solutions increased to 8, 10 and 10 out of the first 10 targets, respectively (Fig 4B, see also Figures C, F in S1 File). The results were further analyzed with respect to fraction of native contacts (*f*_nat_) of near –native docking solutions and the maximum quality of predicted docking geometries (Fig 4). Also for these measures and in case of the protocol with 2x10^6^ MC steps the WTE-H-REMC protocol achieves overall the best performance (Fig 4A). For the extended protocol with 10^7^ MC steps the quality of solutions in terms of *f*_nat_ is more similar for all 4 protocols (Fig 4B), indicating that indeed the standard MC technique requires longer searches to achieve convergence compared to the WTE-H-REMC method. The best *f*_nat_ for protocols MC, REMC and WTE-REMC all increased on average around 17%, getting close to that of the WTE-H-REMC protocol. The best *f*_nat_ for protocol WTE-H-REMC also increased slightly (~7%, Fig 4B). Fig 3 presents two representative examples on targets 1EAW and 3SGQ of ligand RMSD (L_rmsd) versus interaction score (I_sc). Comparing these data with that from the trajectory of 2x10^6^ MC steps, it shows that the details of the energy landscape sampled by WTE-H-REMC remains similar indicating reasonable convergence within 2x10^6^ MC steps for most cases. Meanwhile, the rigid-body space reached for target 1EAW by protocols MC, REMC and WTE-REMC, and on target 3SGQ by protocol MC, drastically improved (Fig 3).

**Fig 5:**
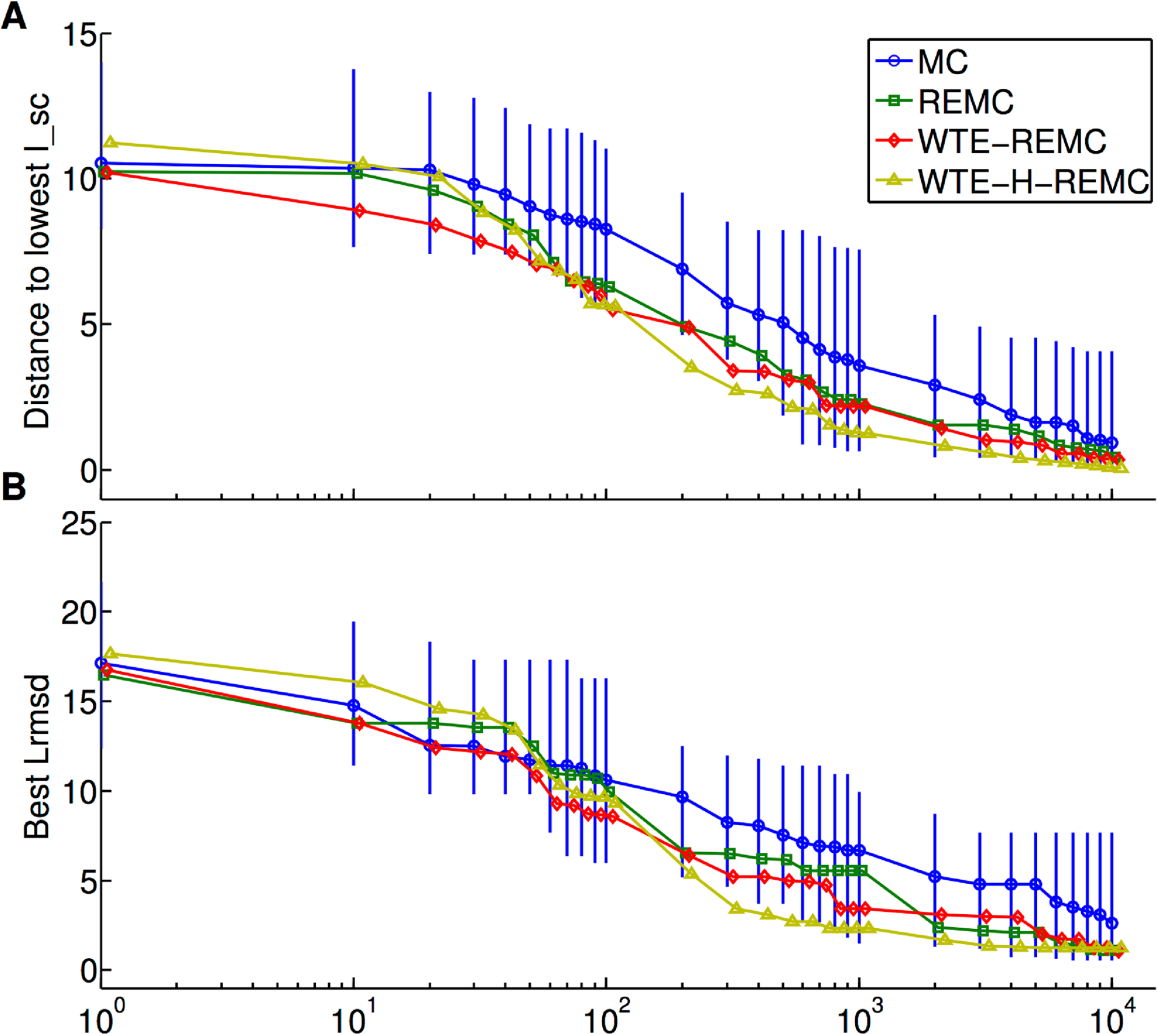
Evolution of I_sc docking interaction score (A) and best sampled Lrmsd (B) vs. MC step number. The MC step number is scaled by x1000. For the interaction score I_sc the smallest difference (sampled up to the selected step number) relative to the lowest scoring complex sampled in the entire docking search is plotted. The variance in sampled scores (up to the considered number of MC steps) is indicated by error bars for the MC protocol. It is of similar magnitude for the other protocols (not shown). For (B) the smallest sampled L_rmsd up to the step number indicated in the x-axis is shown.

Since the force field score is the quantity which is optimized during the docking searches (and not the agreement with the bound structure) it is of interest to compare the protocols in terms of the efficiency to optimize the force field score. For each target, the average of the lowest 10 interaction scores (lowest I_sc) sampled up to a given MC step number was considered (using the extended trajectories) and the difference relative to the lowest score found during the search was recorded. The average of this quantity for all 20 cases was calculated and plotted in Fig 5. The enhanced sampling techniques REMC, WTE-REMC and WTE-H-REMC, consistently, reached lower interaction scores than the standard MC method for a given number of MC step (Fig 5A). The WTE-H-REMC technique reached on average lower I_sc than the other three protocols already after ~3x10^5^ MC steps. Interestingly, the same analysis using the L_rmsd instead of the I_sc yields the same trend, indicating that on average the I_sc score correlates with the L_rmsd (Fig 5B). Low L_rmsd of sampled geometries gives on average (but not for all targets) also a favorable score.

### Protocol testing

A subset of three complexes (pdb-entries: 1PPE, 2OUL and 2SIC) was chosen as an independent protocol test set. For two of these three complexes, only the MC and the WTE_H_REMC protocol had been tested in the main work. The four protocols were run using the RosettaScripts interface with parameters as described above, trajectory length of 2x10^6^ MC steps and a newly created starting conformation. In contrast to the previous docking runs, only a single starting conformation was generated for each complex. All the protocols could be successfully executed. A summary of the test results can be found in Table 2. For all three test cases, the enhanced sampling methods yielded structures of lower L_rmsd and higher *f*_nat_ than standard MC sampling. Enhanced sampling methods generated near-native docking models, whereas standard MC sampling did not yield any structures of CAPRI one star quality or better. The WTE_H_REMC technique was the only method to generate CAPRI three star quality structures for all three cases and thus yielded the best performance on the test set. The results match the previously presented data for these three complexes and thus confirm that the sampling does not depend on the choice of the starting conformation. The randomly generated starting conformations sometimes contained clashes, but the enhanced sampling methods were able to refine them to high-quality solutions. Hence, it might be possible to use enhanced sampling methods also for refinement of docked complexes using other methods than *RosettaDock*.

**Table 2:** Results for protocol testing on a subset of three complexes.

PDB ID	MC	REMC	WTE-REMC	WTE-H-REMC
1PPE	5.6 Å	1.5 Å	2.2 Å	1.0 Å
	14.5 h	14 h	12.3 h	17.8 h
2OUL	4.2 Å	1.9 Å	2.2 Å	0.7 Å
	21.8 h	18.3 h	18 h	22 h
2SIC	4.9 Å	0.4 Å	2.8 Å	0.5 Å
	25.5 h	22 h	19 h	20 h

For all complexes, the best sampled L_rmsd and the execution time on 27 threads are listed.

## Conclusions

In this work, four different Monte Carlo advanced sampling protocols implemented in *RosettaDock* to predict the geometry of protein-protein complexes have been compared. For all the protocols (on each target) the same initial protein-protein docking start configurations were used with 15 Å translational displacements and 60° rotation of one partner from the native complex structure. This situation corresponds to the frequent scenario that the interaction region between proteins is approximately known and start configurations are placed close to the approximately known binding region. It is also very useful for directly comparing the docking performance of different approaches at the computational demanding atomistic high resolution level. Note, that the protocol is computationally too demanding for routine applications that require to search over the entire surface of two protein partners. If complete protein surfaces are considered it is also very likely that the scoring function is not accurate enough to pick out near-native solutions as lowest energy complexes. Our results on docking refinement show indeed that the application of advanced sampling schemes improves the docking refinement performance yielding final configurations in better agreement with the bound structure and yield also a much larger fraction of near-native structures compared to regular MC searches. The WTE-H-REMC consistently gave the best performance since it explores the phase space more efficiently due to larger energy fluctuation and due to the added biasing potential that effectively smoothes the landscape and increases the replica exchange rates. An increase of the number of MC steps to 10^7^ in the standard MC protocol resulted in improved performance achieving a similar fraction of native contacts of best sampled solutions and similar final docking scores compared to WTE-H-REMC with 2x10^6^ steps. Hence, in most cases a standard MC protocol requires roughly 5 times larger computational demand to achieve the same final docking prediction performance. It should be emphasized that this reflects only a general trend. For some test cases even 10^7^ MC steps still gave inferior docking results compared to WTE-H-REMC and still the fraction of the best solutions relative to the total number of sampled geometries is much smaller than for the advanced sampling method. Further improvement might be possible by an adjustment of the bin size in the WTE to collect the history dependent bias energy. However, an even larger gain could be achieved by an improvement of the docking scoring function to increase the gap between ranking near-native and non-native solutions.

## Supporting Information

S1 File Supporting Figures. **Figure A. Scatter plot of interaction score (I_sc) vs. ligand RMSD (L_rmsd) for the first 10 targets after 3x10** ^**5**^ **MC steps.** All the panels have the same L_rmsd range of [0.30], and the same I_sc range of [-15.0 Rosetta score units]. For each target, the tested protocols are grouped together and the corresponding protocol is indicated in the score-axis label on the left side. The snapshot number is color-coded, with dark blue and dark red dots corresponding to decoys sampled at the beginning and towards the end of the sampling interval, respectively. **Figure B. Same as Figure A in S1 File but for the docking searches up to 2x10** ^**6**^ **MC steps. Figure C. Same as Figure A in S1 File but for the docking refinement simulation with 10** ^**7**^ **MC steps. Figure D. Scatter plot of interaction score (I_sc) vs. ligand RMSD (L_rmsd) for the additional 10 targets with 3x10** ^**5**^ **MC steps.** All the panels have the same L_rmsd range of [0.30], and the same I_sc range of [-15.0]. For each target, the tested protocols are grouped together and the corresponding protocol is indicated in the score-axis label on the left side. The snapshots number is color-coded, with dark blue and dark red dots corresponding to decoys sampled at the beginning and the end, respectively. **Figure E. Same as Figure D in S1 File but for docking searches up to 2x10** ^**6**^ **MC steps. Figure F. Same as Figure D in S1 File but for docking searches with 10** ^**7**^ **MC steps.**
(PDF)
