# Supplementary material for: Application of Enhanced Sampling Monte Carlo Methods for High-Resolution Protein-Protein Docking in Rosetta
Source: PLoS One. 2015 Jun 8;10(6):e0125941. doi: 10.1371/journal.pone.0125941 (PMC4459952; doi:10.1371/journal.pone.0125941)
Supplement: S1 File — Figure A. Scatter plot of interaction score (I_sc) vs. ligand RMSD (L_rmsd) for the first 10 targets after 3x10 5 MC steps. All the panels have the same L_rmsd range of [0.30], and the same I_sc range of [-15.0 Rosetta score units]. For each target, the tested protocols are grouped together and the corresponding protocol is indicated in the score-axis label on the left side. The snapshot number is color-coded, with dark blue and dark red dots corresponding to decoys sampled at the beginning and towards the end of the sampling interval, respectively. Figure B. Same as Figure A in S1 File but for the docking searches up to 2x10 6 MC steps. Figure C. Same as Figure A in S1 File but for the docking refinement simulation with 10 7 MC steps. Figure D. Scatter plot of interaction score (I_sc) vs. ligand RMSD (L_rmsd) for the additional 10 targets with 3x10 5 MC steps. All the panels have the same L_rmsd range of [0.30], and the same I_sc range of [-15.0]. For each target, the tested protocols are grouped together and the corresponding protocol is indicated in the score-axis label on the left side. The snapshots number is color-coded, with dark blue and dark red dots corresponding to decoys sampled at the beginning and the end, respectively. Figure E. Same as Figure D in S1 File but for docking searches up to 2x10 6 MC steps. Figure F. Same as Figure D in S1 File but for docking searches with 10 7 MC steps. (PDF) [file pone.0125941.s001.pdf]

Supporting Information

Application of enhanced sampling Monte Carlo methods for high-resolution protein-protein docking in Rosetta

Zhe Zhang, Christina E.M. Schindler, Oliver F. Lange and Martin Zacharias

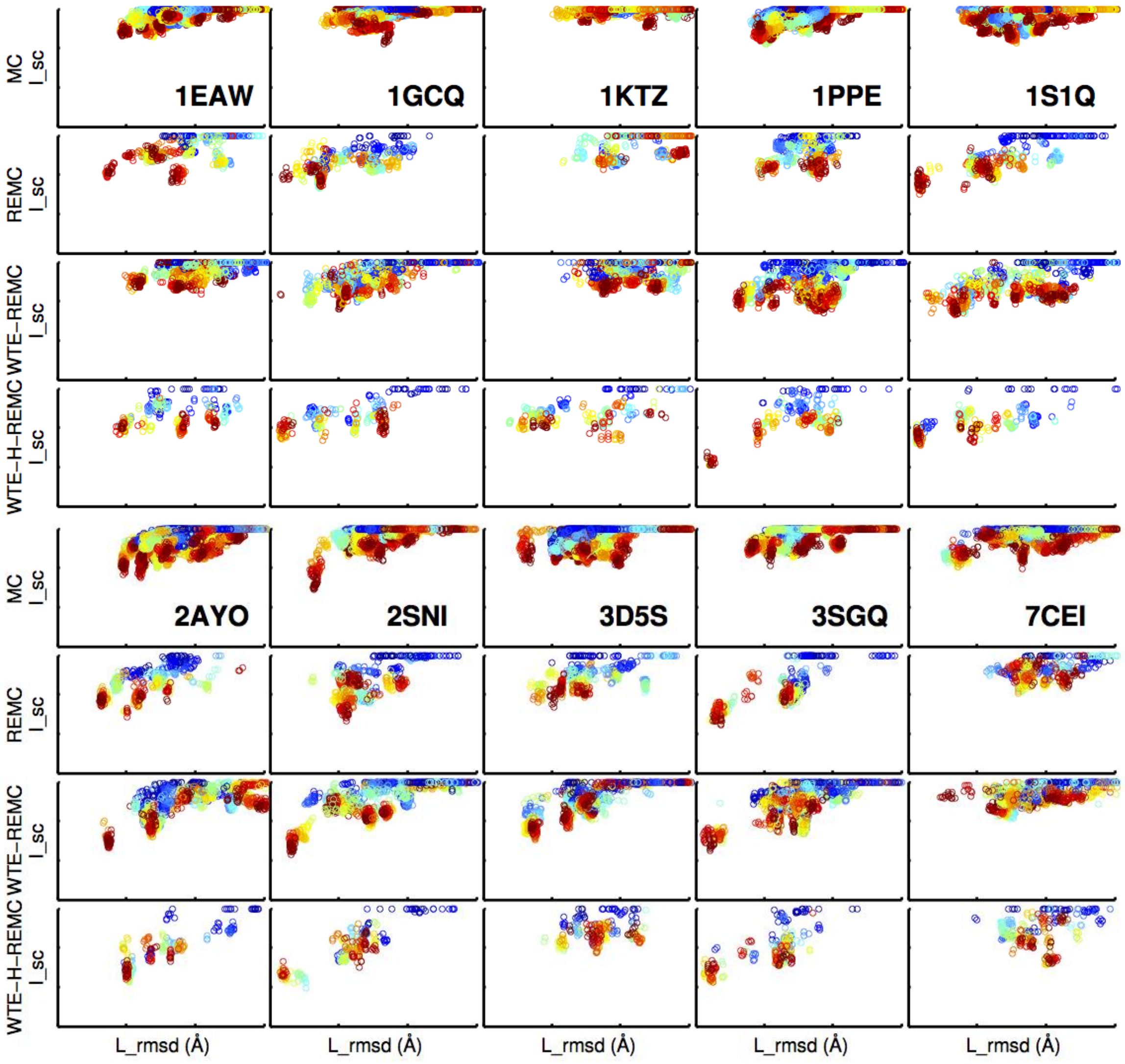

Figure A.

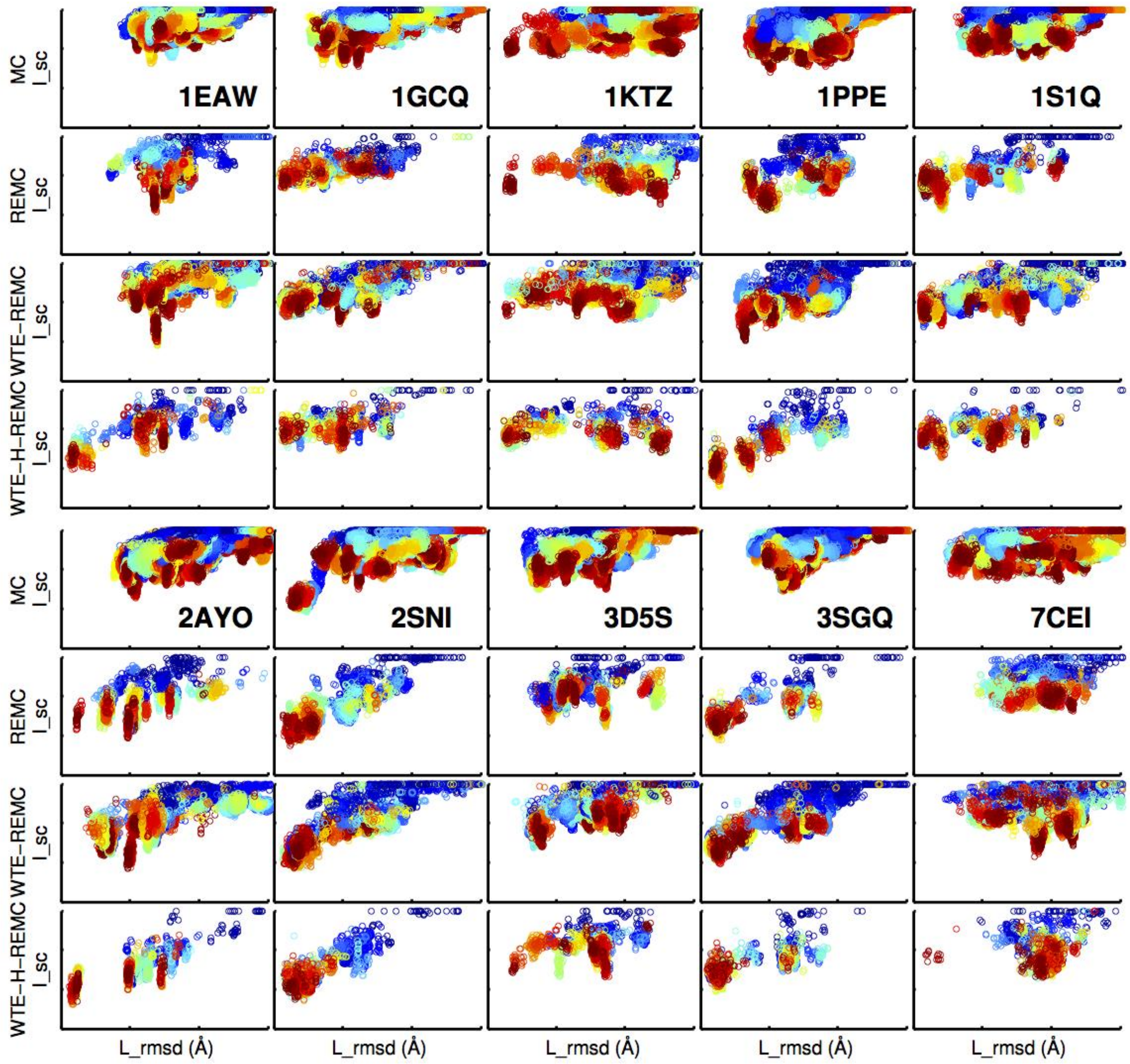

MC
REMC
WTE−REMC
WTE−H−REMC
I_sc
1EAW
1GCQ
1KTZ
1PPE
1S1Q
2AYO
2SNI
3D5S
3SGQ
7CEI
L_rmsd (Å)

Figure B.

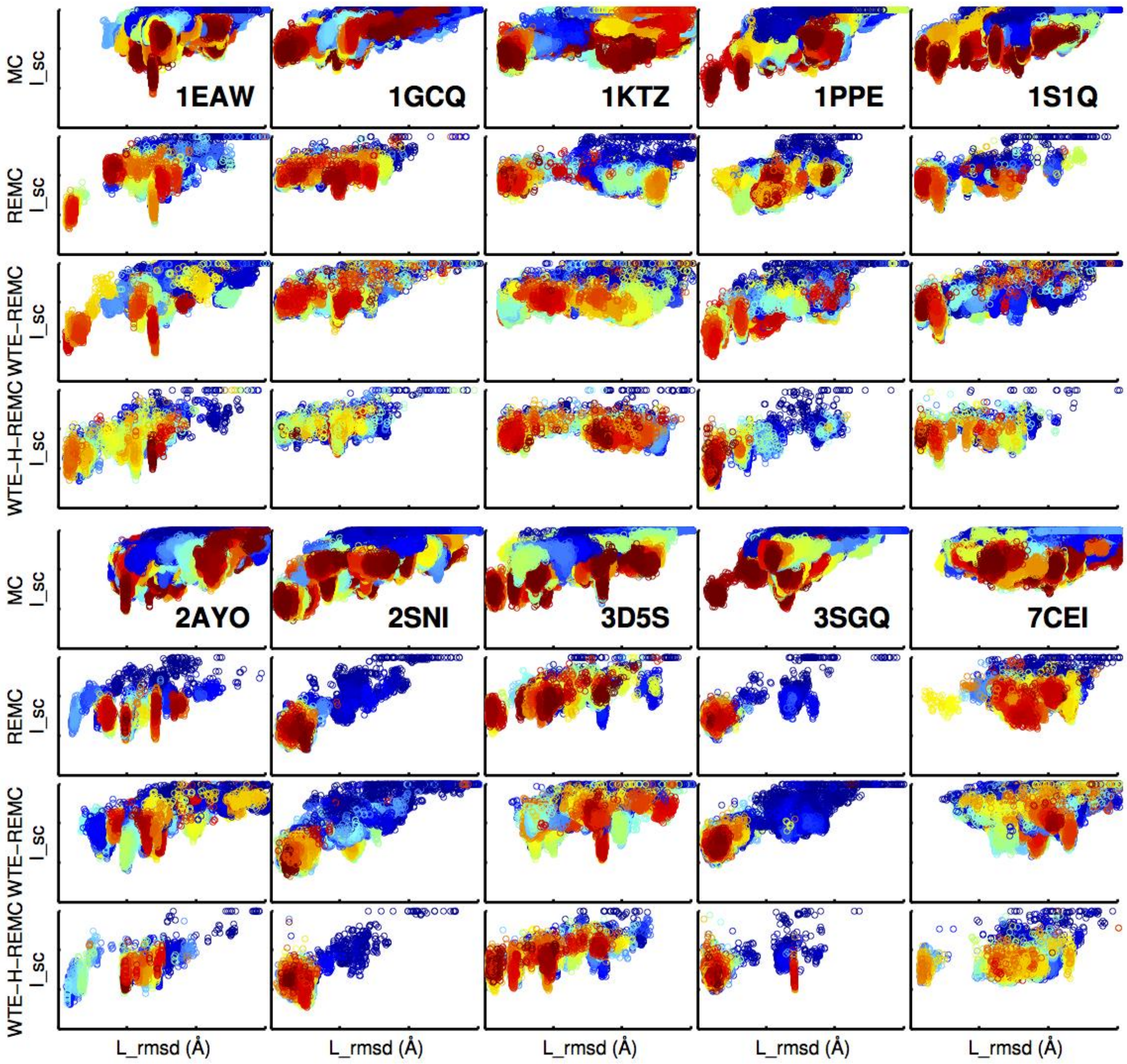
MC
I_sc
REMC
I_sc
WTE−REMC
I_sc
WTE−H−REMC
I_sc
1EAW
1GCQ
1KTZ
1PPE
1S1Q
2AYO
2SNI
3D5S
3SGQ
7CEI
L_rmsd (Å)
L_rmsd (Å)
L_rmsd (Å)
L_rmsd (Å)
L_rmsd (Å)

Figure C.

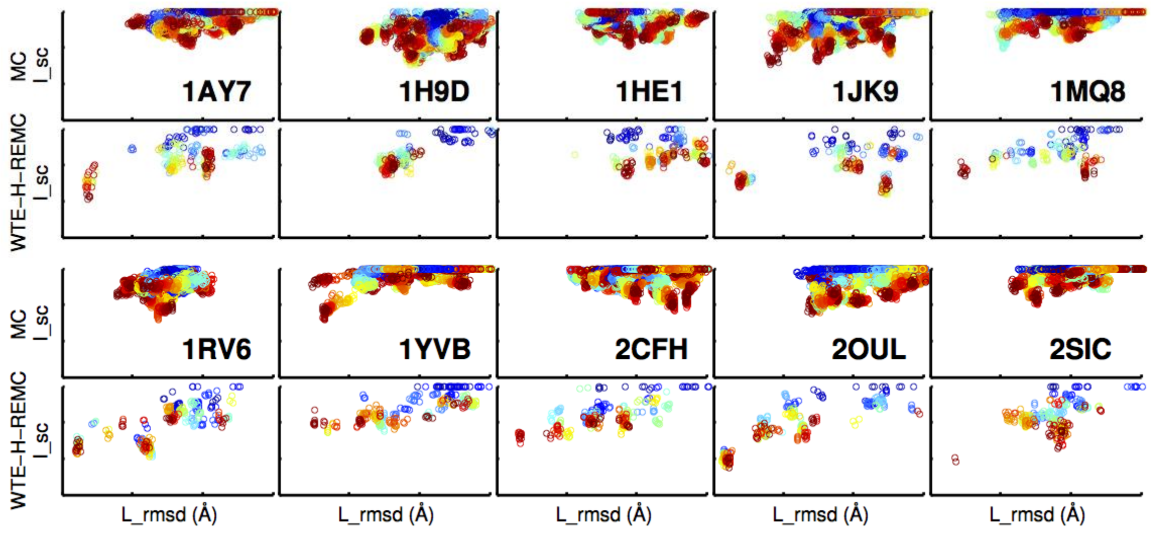
MC
I_sc
WTE–H–REMC
I_sc
1AY7
1H9D
1HE1
1JK9
1MQ8
1RV6
1YVB
2CFH
2OUL
2SIC
L_rmsd (Å)
L_rmsd (Å)
L_rmsd (Å)
L_rmsd (Å)
L_rmsd (Å)

Figure D.

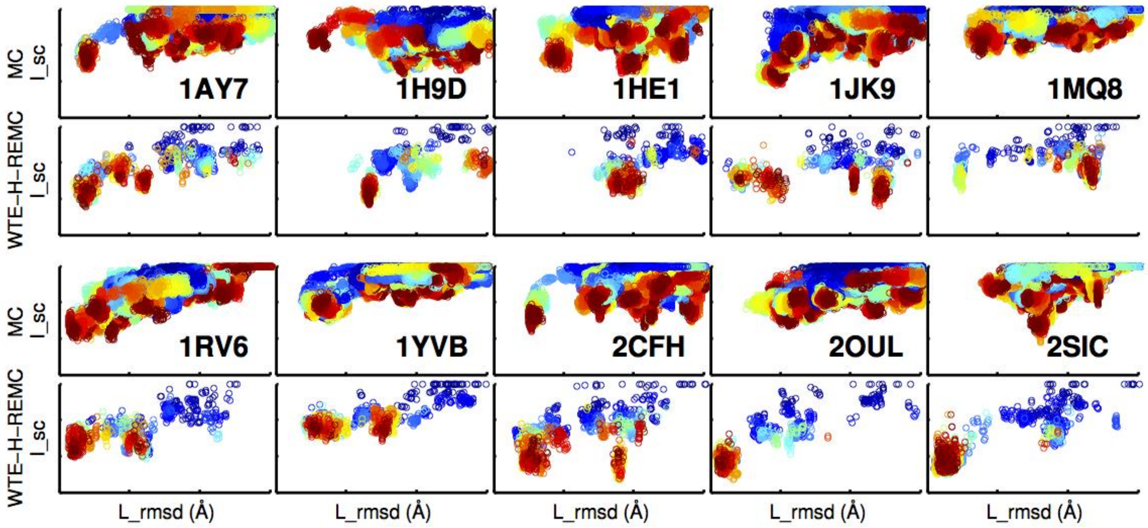

MC
I_sc
WTE-H-REMC
I_sc
1AY7
1H9D
1HE1
1JK9
1MQ8
1RV6
1YVB
2CFH
2OUL
2SIC
L_rmsd (Å)

Figure E.

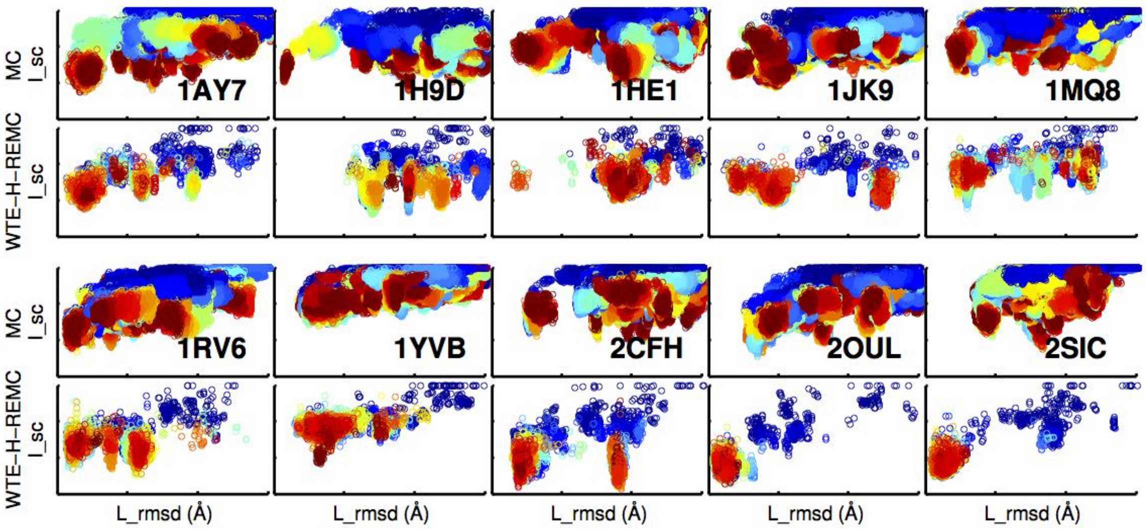

MC
l_sc
WTE–H–REMC
l_sc
1AY7
1H9D
1HE1
1JK9
1MQ8
1RV6
1YVB
2CFH
2OUL
2SIC
L_rmsd (Å)

Figure F.
